# Commentary: PET/CT radiomics–biomarker diagnostic model for identifying lymphoma in fever of unknown origin

**DOI:** 10.3389/fimmu.2026.1888226

**Published:** 2026-07-20

**Authors:** Lei Tang, Ling Liang, Ji Ma

**Affiliations:** 1 Department of Radiology, Suining Central Hospital, Suining, China; 2 Department of Ultrasonography, Suining Central Hospital, Suining, China; 3 Department of Oncology, Suining Central Hospital, Suining, China

**Keywords:** biomarkers, fever of unknown origin, lymphadenopathy, lymphoma, PET/CT radiomics

## 1 The diagnostic dilemma of FUO with lymphadenopathy

Fever of unknown origin (FUO) remains one of the most challenging clinical syndromes in modern medicine because of its broad and heterogeneous etiological spectrum, encompassing infectious diseases, malignancies, autoimmune disorders, and other inflammatory conditions (1). The diagnostic complexity increases substantially when FUO is accompanied by lymphadenopathy. In this setting, malignant lymphoma, adult-onset Still’s disease, necrotizing lymphadenitis, and certain infectious disorders may all present with persistent fever, elevated inflammatory markers, enlarged lymph nodes, and increased fluorodeoxyglucose (FDG) uptake on PET/CT imaging. Consequently, significant overlap exists among these entities in terms of clinical manifestations, laboratory findings, and metabolic imaging characteristics, often making early and accurate differentiation extremely difficult.

^18^F-FDG PET/CT has become an indispensable imaging modality in the diagnostic workup of FUO because of its ability to detect metabolically active lesions throughout the entire body and guide subsequent biopsy or therapeutic decision-making. Nevertheless, the specificity of PET/CT in distinguishing malignant from benign lymphadenopathy remains limited. Inflammatory and immune-mediated lymph node diseases may demonstrate FDG avidity comparable to that observed in lymphoma, thereby reducing the discriminative value of conventional metabolic parameters such as the maximum standardized uptake value (SUVmax). Against this background, the recent study by Jing et al. published in Frontiers in Immunology proposed a multimodal diagnostic model integrating PET/CT radiomics and circulating inflammatory biomarkers to improve the differentiation between lymphoma and benign lymphadenopathy in patients with FUO (2).

## 2 From conventional PET metrics to radiomics-based analysis

One of the major strengths of this study lies in its departure from the traditional reliance on single metabolic parameters. Although SUVmax is widely used in routine PET/CT interpretation, it reflects only the highest metabolic voxel within a lesion and fails to capture the complex intralesional heterogeneity that often characterizes malignant tumors. Inflammatory diseases associated with FUO, particularly adult-onset Still’s disease and necrotizing lymphadenitis, may also exhibit markedly increased FDG uptake because of intense inflammatory cell infiltration and activated immune responses. As a result, SUVmax alone is frequently insufficient for reliable differential diagnosis.

Radiomics offers a promising solution to this limitation by enabling high-throughput extraction of quantitative imaging features from PET and CT data (3–5). These features include morphological descriptors, histogram-based parameters, textural indices, and spatial heterogeneity metrics, all of which may reflect underlying biological characteristics such as cellular density, necrosis, metabolic variability, and tissue architectural disruption. In lymphoma, clonal proliferation and heterogeneous tumor microenvironments may generate distinctive radiomic signatures that differ from those observed in benign inflammatory lymphadenopathy.

In the study by Jing et al., radiomics features derived from both PET and CT images underwent dimensionality reduction using least absolute shrinkage and selection operator (LASSO) regression, resulting in the selection of six PET features and four CT features for model construction. The authors subsequently established PET radiomics scores (PRSCORE), CT radiomics scores (CRSCORE), and an integrated radiomics score (RSCORE). This design highlights the complementary nature of PET and CT information: PET characterizes metabolic heterogeneity, whereas CT provides structural and anatomical detail. Their integration therefore allows a more comprehensive characterization of lymph node lesions than either modality alone.

## 3 Integrating systemic biomarkers with local imaging features

Another notable aspect of the study is the incorporation of inflammatory biomarkers into the diagnostic framework. FUO is fundamentally a systemic syndrome rather than an isolated local disease process. Therefore, relying solely on imaging characteristics may not fully reflect the underlying pathophysiological state.

The authors incorporated procalcitonin (PCT) and serum amyloid A (SAA) into the predictive model and ultimately identified PCT, SAA, and RSCORE as independent predictors in the final combined model. Importantly, these biomarkers do not merely serve as supplementary variables; instead, they provide complementary biological information distinct from imaging-derived heterogeneity. Radiomics captures the spatial and metabolic complexity of local lesions, whereas circulating biomarkers reflect systemic inflammatory and immune responses. The integration of these two dimensions represents a biologically meaningful multimodal strategy.

Importantly, these two biomarkers represent complementary but biologically distinct inflammatory pathways: PCT primarily reflects acute infection-associated innate immune activation, whereas SAA is more closely linked to prolonged systemic inflammatory and cytokine-driven responses. This biological divergence explains why their combined inclusion improves model robustness rather than introducing redundant information.

The biological relevance of PCT and SAA warrants particular attention. PCT is traditionally regarded as an indicator of bacterial infection and systemic inflammatory activation, whereas SAA is a highly sensitive acute-phase reactant associated with inflammatory burden. Although both lymphoma and inflammatory disorders can elevate these markers, the patterns and combinations of biomarker expression may differ substantially between malignant and benign conditions. By combining systemic inflammatory signatures with lesion-level radiomic heterogeneity, the model provides a more holistic representation of disease biology.

## 4 Diagnostic performance and clinical implications

The study included 203 patients with FUO-associated lymphadenopathy, comprising 114 cases of lymphoma and 89 benign lymph node disorders. The multimodal combined model demonstrated excellent diagnostic performance, with an area under the receiver operating characteristic curve (AUC) of 0.970 in the training cohort and 0.958 in the testing cohort, outperforming both the biomarker-only and radiomics-only models.

These findings suggest that multimodal data fusion may significantly improve the differential diagnosis of FUO-related lymphadenopathy. Clinically, such an approach could have several important implications. First, it may facilitate earlier identification of patients at high risk for lymphoma, thereby accelerating definitive diagnosis and treatment initiation. Second, the model may assist clinicians in selecting the most appropriate lymph nodes for biopsy, an issue of considerable practical importance in patients with multifocal lymphadenopathy. Third, quantitative risk stratification may help reduce unnecessary invasive procedures in patients with low predicted malignant risk.

More broadly, this work reflects an important conceptual transition in diagnostic imaging—from qualitative visual interpretation toward data-driven precision medicine. Rather than relying exclusively on isolated imaging signs or laboratory abnormalities, future diagnostic frameworks are increasingly likely to incorporate multidimensional datasets, including radiomics, clinical variables, molecular biomarkers, and potentially genomic information.

## 5 Limitations and remaining challenges

Despite its promising results, several limitations should be acknowledged. First, the study was retrospective and conducted at a single center, raising the possibility of selection bias and limiting generalizability across different patient populations and imaging platforms. Second, although the combined model achieved the highest AUC in both cohorts, the differences between models in the testing cohort did not reach statistical significance, suggesting that the incremental diagnostic benefit requires validation in larger external datasets.

Third, radiomics feature extraction depended on manual delineation of volumes of interest (VOIs), which inevitably introduces interobserver variability despite the involvement of experienced nuclear medicine physicians. Furthermore, radiomics features may be influenced by scanner type, image acquisition protocols, reconstruction algorithms, and preprocessing methods, all of which remain major barriers to widespread clinical implementation.

Another important issue concerns clinical interpretability. Although the nomogram developed in the study provides individualized probability estimation, the practical application of such models in real-world clinical workflows remains uncertain. For instance, it remains unclear which risk thresholds should trigger immediate biopsy, short-term surveillance, or further diagnostic testing. Therefore, future studies should focus not only on statistical performance metrics such as AUC, but also on clinical utility, decision-making impact, and cost-effectiveness.

## 6 Future perspectives

Future investigations should prioritize large-scale multicenter prospective validation to assess the robustness and reproducibility of multimodal diagnostic models across diverse clinical settings. Standardization of radiomics workflows—including image acquisition, segmentation, feature extraction, and reporting—will be essential for broader clinical adoption.

In addition, integration of emerging molecular biomarkers, such as circulating tumor DNA (ctDNA), microRNAs, cytokine profiles, and immune signatures, may further enhance diagnostic precision. Ultimately, the convergence of radiomics, molecular diagnostics, and artificial intelligence may enable the development of truly integrated precision diagnostic systems for FUO and other complex clinical syndromes.

These standardized components collectively represent the minimum methodological requirements for ensuring reproducibility, reducing inter-institutional variability, and facilitating the translation of radiomics-based models from retrospective research settings into clinically deployable decision-support tools.

## 7 Conclusion

The study by Jing et al. represents a valuable step toward improving the differential diagnosis of FUO-associated lymphadenopathy through multimodal integration of PET/CT radiomics and inflammatory biomarkers. Its significance extends beyond the development of a high-performing predictive model; more importantly, it exemplifies the ongoing transformation of diagnostic medicine from single-parameter assessment toward multidimensional data integration. With continued external validation, methodological standardization, and refinement of clinical decision pathways, PET/CT radiomics–biomarker models may become an important component of precision diagnostic strategies for FUO in the future.
